# The microRNA-7-mediated reduction in EPAC-1 contributes to vascular endothelial permeability and eNOS uncoupling in murine experimental retinopathy

**DOI:** 10.1007/s00592-017-0985-y

**Published:** 2017-03-28

**Authors:** Veronica Garcia-Morales, Julian Friedrich, Lysanne M. Jorna, Manuel Campos-Toimil, Hans-Peter Hammes, Martina Schmidt, Guido Krenning

**Affiliations:** 1 Group of Research in Pharmacology of Chronic Diseases (CDPHARMA), Center for Research in Molecular Medicine and Chronic Diseases (CIMUS), University of Santiago de Compostela, 15782 Santiago de Compostela, Spain; 2 International Research and Training Network on Diabetic Microvascular Complications (GRK1874/DIAMICOM), University of Heidelberg, Heidelberg, Germany; 3 International Research and Training Network on Diabetic Microvascular Complications (GRK1874/DIAMICOM), University Medical Center Groningen, Groningen, The Netherlands; 4 Cardiovascular Regenerative Medicine (CAVAREM), Department of Pathology and Medical Biology, University Medical Center Groningen, University of Groningen, Hanzeplein 1 (EA11), 9713 GZ Groningen, The Netherlands; 5 Section of Endocrinology, 5th Medical Department, Medical Faculty Mannheim, University of Heidelberg, Heidelberg, Germany; 6 Department of Molecular Pharmacology, University of Groningen, Groningen, The Netherlands

**Keywords:** EPAC-1, Hypoxia, MicroRNA-7, Endothelial cell, Retinopathy

## Abstract

**Aims:**

To investigate the consequences of oxidative stress and hypoxia on EPAC-1 expression during retinopathy.

**Methods:**

Oxygen-induced retinopathy was induced in mice and EPAC-1 expression investigated by immunofluorescence. In silico analyses were used to identify a link between EPAC-1 expression and microRNA-7-5p in endothelial cells and confirmed by western blot analyses on cells expressing microRNA-7-5p. In vitro, endothelial cells were either incubated at 2% oxygen or transfected with microRNA-7-5p, and the effects of these treatments on EPAC-1 expression, endothelial hyperpermeability and NO production were assessed. In the Ins2Akita mouse model, levels of EPAC-1 expression as well as microRNA-7-5p were assessed by qPCR. Endothelial nitric oxide synthase was assessed by immunoblotting in the Ins2Akita model.

**Results:**

Hypoxia induces the expression of microRNA-7-5p that translationally inhibits the expression of EPAC-1 in endothelial cells, resulting in hyperpermeability and the loss of eNOS activity. Activation of EPAC-1 by the cAMP analogue 8-pCPT-2′-*O*-Me-cAMP reduced the sensitivity of EPAC-1 to oxidative stress and restored the endothelial permeability to baseline levels. Additionally, 8-pCPT-2′-*O*-Me-cAMP rescued eNOS activity and NO production. In mouse models of retinopathy, i.e., oxygen-induced retinopathy and the spontaneous diabetic heterozygous Ins2^Akita^ mice, EPAC-1 levels are decreased which is associated with an increase in microRNA-7-5p expression and reduced eNOS activity.

**Conclusion/Interpretation:**

In retinopathy, EPAC-1 expression is decreased in a microRNA-7-mediated manner, contributing to endothelial dysfunction. Pharmacological activation of remnant EPAC-1 rescues endothelial function. Collectively, these data indicate that EPAC-1 resembles an efficacious and druggable target molecule for the amelioration of (diabetic) retinopathy.

**Electronic supplementary material:**

The online version of this article (doi:10.1007/s00592-017-0985-y) contains supplementary material, which is available to authorized users.

## Introduction

The vascular endothelium exhibits multiple structural and functional abnormalities in response to hypoxia that may contribute to the pathogenesis of several vascular diseases, including (diabetic) retinopathy [1]. Hypoxia is associated with an increment in oxidative stress [2] and the disruption of endothelial adhesion molecules [3, 4], resulting in increased endothelial permeability [5] and impairment of vasodilation [6, 7].

3′,5′-Cyclic adenosine monophosphate (cAMP) is an ubiquitous second messenger that activates two downstream signaling cascades, i.e., protein kinase A (PKA) and the more recently discovered exchange protein activated by cAMP (EPAC) [8, 9]. In endothelial cells, EPAC signaling enhances the barrier function by promoting VE-cadherin junctional stability, thereby reducing endothelial permeability [10, 11]. Corroboratively, Epac1 activation by cAMP or the cAMP analogue 8-pCPT reverses endothelial hyperpermeability induced by inflammatory mediators [12, 13]. Next to the regulatory effects on the endothelial barrier, EPAC participates in the cAMP-induced vascular relaxation in arteries [14, 15], in part by activating endothelial nitric oxide synthase (eNOS) [16, 17]. Concurringly, EPAC expression is dysregulated in pathologies that are characterized by endothelial dysfunction and edema formation [18–20].

Although the downstream consequences of EPAC activation on endothelial function receive increasing attention [20], factors regulating the expression of EPAC during pathology remain elusive. In retinopathy hypoxia might contribute to EPAC dysregulation. We recently uncovered that EPAC-1 is targeted by microRNA-7 in human pulmonary smooth muscle cells, and microRNA-7 expression is associated with increased oxidative stress levels [21]. Therefore, we hypothesized that microRNA-7 might induce EPAC-1 deregulation during retinal hypoxia or in diabetic conditions.

Here, we describe that EPAC-1 expression is inhibited by hypoxia in vivo in the oxygen-induced retinopathy mouse model [22, 23] and in endothelial cell cultures exposed to hypoxia. Furthermore, we show that the reduction in EPAC-1 expression is associated with the hypoxia-induced expression of microRNA-7, resulting in translational repression. Activation of the remnant EPAC-1 in endothelial cells counteracts hypoxia-induced endothelial hyperpermeability and reverses the NO/ROS imbalance through eNOS activation. Moreover, in the Ins2^Akita^ mouse model for diabetic retinopathy (DR), EPAC-1 expression is vastly reduced, which coincides with a marked increase in microRNA-7 expression. These data indicate that EPAC-1 is a pivotal regulator of endothelial function in (diabetic) microangiopathies involving endothelial dysfunction associated with hypoxia, and might serve as promising therapeutic targets to ameliorate these conditions.

## Animals, materials and methods

### Animals and ethical approval

C57BL/6J mice and spontaneous diabetic heterozygous Ins2Akita^+/−^ mice (Jackson Laboratory, Charles River, Sulzfeld, Germany) were used throughout the study. Age-matched non-diabetic homozygous Ins2Akita^−/−^ mice served as control. All experimental procedures were performed according to the guidelines of the statement for animal experimentation issued by the Association for Research in Vision and Opthalmology and were approved by the local board for animal care (Medical Faculty Mannheim, Germany).

### Oxygen-induced retinopathy mouse model

Oxygen-induced retinopathy was induced in C57BL/6J mice as described previously [22, 23]. In short, newborn mice (*n* = 6) at postnatal day (p) 7 were exposed to hyperoxia (75% oxygen) in an incubation chamber (Stuart Scientific, Redhill, UK) with their nursing mothers for 5 days and then returned to ambient air, creating a relative hypoxic environment. Control mice (*n* = 6) were kept at ambient air and used as a control group. Mice were killed at p12 (i.e., 6 h of relative hypoxia) and p13 (i.e., 24 h of relative hypoxia), and the retinas were isolated as described previously [23].

### Retinopathy in Ins2Akita mice

After 6 months of diabetes, mice (*n* = 6/group) were killed and the retinas were isolated as described previously [23].

### Human endothelial cell culture

Human umbilical vein endothelial cells (Lonza, Breda, the Netherlands) were cultured in endothelial growth medium, consisting of RPMI 1640, l-glutamine (2 mM), penicillin/streptomycin (1%; all Lonza, Breda, the Netherlands), bovine pituitary extract (20 µg/ml; Invitrogen/Life Technologies, Bleiswijk, The Netherlands), heparin (5 U/ml; Leo Pharma, Amsterdam, The Netherlands), and FBS (20%; Thermo Scientific, Waltham, MA).

When appropriate, confluent endothelial cell cultures were serum starved for 24 h, and cells were treated with fenoterol (1 µM; Boehringer Ingelheim, Germany), forskolin (10 µM; Tocris, UK), 6-Bnz-cAMP (300 µM), 8-pCPT-2′-*O*-Me-cAMP (100 µM) (Biolog Life Science Institute, Germany), or ESI-09 (5 µM, SelleckChem, Germany).

Parallel cultures were maintained under normoxic (21% oxygen tension) and hypoxic conditions (2% oxygen tension) for 48 h prior to experiments. To establish hypoxia, cell culture medium was deoxygenated by bubbling gaseous N_2_ through the medium at room temperature for 30 min. Cells were maintained in an hypoxic cell culture incubator at 37 °C containing 2% O_2_, 93% N_2_, and 5% CO_2_.

Construction of 3′UTR reporter constructs and microRNA-7-5p transfection in COS7 and Endothelial cells. The 3′UTR fragments of EPAC-1 and EPAC-2 were isolated by conventional PCR amplification. 5′ SgfI- and 3′ NotI restriction sites (underlined) were incorporated in the primer sequences; EPAC-1 sense: 5′-CCGCCGGCGATCGCAGGAGTGGGTGGAGAGTGGA-3′ and antisense: 5′-CATGCGGCCGCGTGTCCCCACCCACGGCAAG-3′ and EPAC-2 sense: 5′-ATATATGCGATCGCACATTTCAAATGCCCAAAGC-3′ and antisense: 5′-GCAGCGGCCGCATTGAATGAACTATTTACAA-3′. Amplicons were isolated using the QIAquick Gel Extraction Kit (Qiagen Inc) according to manufacturer’s instructions, modified using SfgI and NotI restriction enzymes (Fermentas) and inserted in the psiCHECK-2 vector (Promega) using T4 ligase.

MicroRNA-7 mimics and scrambled sequences were obtained from ThermoFisher Scientific and co-transfected with 3′UTR reporter constructs into COS-7 cells using EndoFectin (GeneCopoeia, Rockville, MD). Co-transfection of miR-7 mimics and an 3′UTR-free psiCHECK-2 plasmid were used as controls.

Endothelial cells were transfected using microRNA-7 mimics and scrambled sequences using EndoFectin (GeneCopoeia, Rockville, MD).

### Endothelial permeability assays

Endothelial cells (1.0 × 10^5^/cm^2^) were cultured on polycarbonate cell culture inserts (pore size 0.4 µm, porosity 0.9·10^8^/cm^2^; Nunc, ThermoFisher, Waltham, CA) coated with 0.1% gelatin for 48 h. When appropriate, cultures were serum starved for 24 h and cells were treated with fenoterol (1 µM; Boehringer Ingelheim, Germany), forskolin (10 µM; Tocris, UK), 6-Bnz-cAMP (300 µM) or 8-pCPT-2′-*O*-Me-cAMP (100 µM) (Biolog Life Science Institute, Germany). Parallel cultures were maintained under normoxic (21% oxygen tension) and hypoxic conditions (2% oxygen tension) for 48 h prior to experiments. Permeability was assessed by the addition of 10 µg/ml FITC-dextran in the upper compartments, and fluorescence in the lower compartments was assessed on a spectrofluorescence reader at Ex485/Em519 after 30 min.

### Immunofluorescence

Endothelial cells were cultured in eight-well Lab-Tek^®^ chamber slides (Nunc, IL, USA) until 80% confluence was reached. Cells were incubated under normoxic or hypoxic conditions for 24 h. Samples were fixed in 2% paraformaldehyde in PBS for 20 min and permeabilized with 0.3% Triton X-100 for 10 min. Blocking of unspecific antibody activity was performed using in 2% BSA. Samples were incubated with rabbit polyclonal antibodies to human EPAC-1 (Abcam, #ab21236, 1:100) and eNOS (BD #61098, 1:200) diluted in 2%BSA/PBS overnight at 4 °C. Samples were washed and incubated with Alexa Fluor^®^ 594-conjugated goat anti-rabbit IgG antibodies (Molecular Probes, Invitrogen, OR, USA) diluted 1:500 in DAPI/PBS for 1 h at room temperature. Samples were mounted in Citifluor AF1 (Agar Scientific, UK), and cells were examined using a Leica TCS SP8 (Leica Microsystems, Germany) laser scanning fluorescence confocal microscope using a 63x/1.40 oil objective.

### Gene and microRNA transcript analysis

Total RNA was isolated using TRIzol reagent (Invitrogen, Waltham, CA) according to manufacturer’s instructions and quantified by spectrophotometry (NanoDrop Technologies, Waltham, MA). For gene expression analyses, 1 µg of total RNA was reversely transcribed into cDNA using the RevertAid™ First-Strand cDNA synthesis kit (Applied Biosystems, Carlsbad, CA) and amplified using species-specific primers (human primers: Suppl. Table 1; mouse primers Suppl. Table 3). For microRNA expression analyses, 20 ng total RNA was reversely transcribed using the TaqMan MicroRNA Reverse Transcription kit (Applied Biosystems) using specific stemloop templates for miRNA-7-5p (5′-GTCGTATCCAGTGCAGGGTCCGAGGTATTCGCACTGGATACGACACAACAAA-3′) or RNU6 (5′-GTCGTATCCAGTGCAGGGTCCGAGGTATTCGCACTGGATACGACAAAAATATGG-3′) and amplified using sense 5′-TGCGGTTGGAAGACTAGTGAT-3′, antisense 5′-CCAGTGCAGGGTCCGAGGTCCG-3′ for miR-7-5p, and sense 5′-TGCGGCTGCGCAAGGATGA-3′, antisense 5′-CCAGTGCAGGGTCCGAGGTCCG-3′ for RNU6. Quantitative PCR expression analysis was performed on a reaction mixture containing 10 ng cDNA equivalent, 0.5 µM sense primers, and 0.5 µM antisense primers (all Biolegio, Leiden, The Netherlands) and FastStart SYBR Green (Roche, Almere, The Netherlands). Analyses were run on a Viia7 real-time PCR system (Applied Biosystems, Carlsbad, CA). Each reaction was performed in triplicate and gene expression was calculated using the ΔΔCt method. The data are expressed as fold change versus control.

### Protein analysis

Retinal digests and endothelial cells were lysed in RIPA buffer (ThermoScientific, Waltham, MA) and protein concentration determined by DC Protein Assays (BioRad, Hercules, CA) according to manufacturers’ instructions. 30 µg of protein/lane was loaded on a SDS-PAGE gel (8–12%) for electrophoresis and transferred to a nitrocellulose membrane. Membranes were incubated overnight with antibodies to EPAC-1 (Abcam, Cambridge, UK, #ab21236, 1:1000), VE-Cadherin (Cell Signaling, #2500, 1:1000), eNOS (BD, San Jose, CA, #61098, 1:2000), phopho-Ser1177 eNOS (BD, San Jose, CA, #612393, 1:1000), and β-actin (Cell Signaling, #4967, 1:5000) for 1 h at RT. IRDye^®^-labeled antibodies (1:10.000, Li-COR Biosciences, Lincoln, NE) were used for detection. Bands were visualized using the Odissey^®^ Infrared Imaging System (Li-COR Biosciences, NE, USA). Densitometry was performed using Image J version 1.45 s (NIH, Bethesda, MD). Protein expression levels were normalized to β-actin.

### Nitrite and ROS measurements

Nitrite levels in the cell culture medium, a stabile indicator of NO production, were quantified using the Measure-iT™ High-Sensitivity Nitrite Assay Kit (Molecular Probes, Eugene, OR) according to the manufacturer’s protocol. Obtained nitrite concentrations were normalized against the total amount of cellular protein using the DC protein.

Reactive oxygen levels were determined by incubating the cells with 50 µM 2′,7′-dichlorofluorescin diacetate (DCFH-DA, Sigma-Aldrich, St. Louis, MO) for 10 min in dark. Cells were dissociated using Accutase™ solution (PAA Laboratories, Austria), pelleted by centrifugation, and suspended in PBS. The generation of intracellular ROS was determined by flow cytometry on the FACSCalibur and WinList version 6.0 software (both BD Biosciences, CA, USA).

### MicroRNA in situ hybridization

Double DIG-labeled MicroRNA-7-5p and scrambled control probes, microRNA ISH buffers, and reagents were obtained Exiqon (Vedbaek, Denmark) and used according to the manufacturers’ protocol. Hybridization was performed for 16 h at 44 °C in a humidified chamber.

### Data presentation and statistical analysis

Data is expressed as mean ± SEM. Significant differences between two means were determined by Mann–Whitney two-tailed *U* test for unpaired data or by one-way analysis of variance (ANOVA) followed by Dunnett’s post hoc test, where appropriate. *p* values <0.05 were considered statistically significant.

## Results

### Hypoxia decreases EPAC-1 expression

**Fig. 1:**
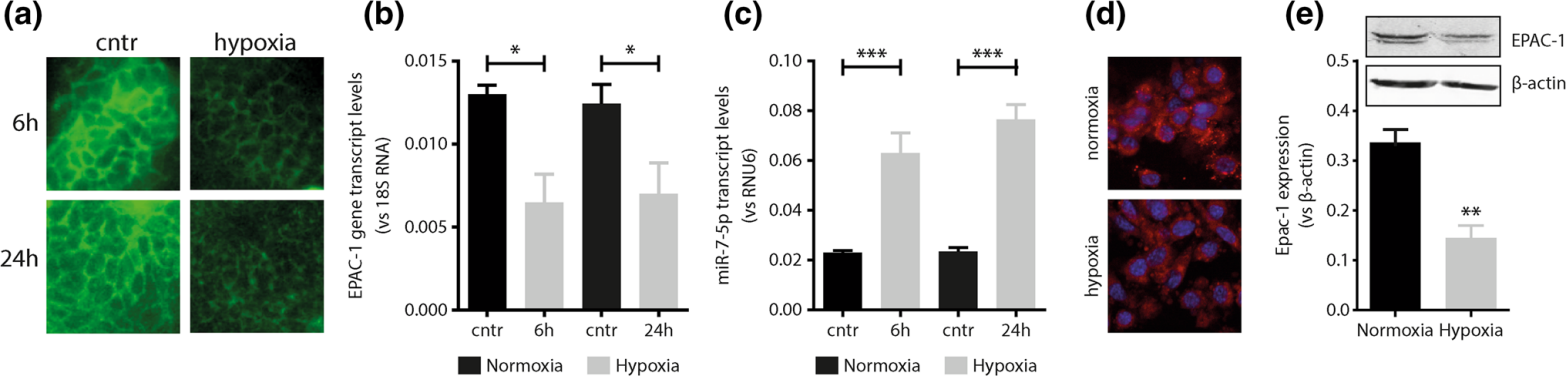
Hypoxia decreases EPAC-1 expression. **a** Immunofluorescence analysis and **b** gene expression analysis for Epac-1 following relative hypoxia in vivo for 6 and 24 h. Hypoxia decreases EPAC-1 in the oxygen-induced retinopathy model. Conversely, **c** hypoxia increases retinal microRNA-7-5p expression at 6 and 24 h. **d** Immunofluorescence analysis and **e** western blot analysis for EPAC-1 in vitro. Long-term (24 h) hypoxia decreases EPAC-1 expression in cultured endothelial cells. Cntr = unstimulated endothelial cells; *p < 0.05; **p < 0.01. Data are expressed as mean ± SEM of at least three independent experiments

Oxygen-induced retinopathy is associated with a marked decrease in EPAC-1 expression (Fig. 1a). After 6 h and 24 h of relative hypoxia, retinal EPAC-1 gene transcript levels were reduced (2.0- and 1.9-fold, respectively, *p* < 0.05, Fig. 1b Conversely, miR-7 expression was increased in the retina of OIR-mice (2.7- and 3.2-fold, *p* < 0.01) compared to normoxic control mice (Fig. 1c). The effect of hypoxia on EPAC-1 expression was confirmed in endothelial cell cultures exposed to 2% (hypoxia) or 20% (normoxia) oxygen, where EPAC-1 protein expression decreased (2.4-fold, *p* < 0.01, Fig. 1d, e) when cells were exposed to hypoxia for 24 h. These data indicate that the expression of EPAC-1 is oxygen sensitive and its expression is decreased during hypoxic stress.

### Hypoxia induces microRNA-7-mediated suppression of EPAC-1

**Fig. 2:**
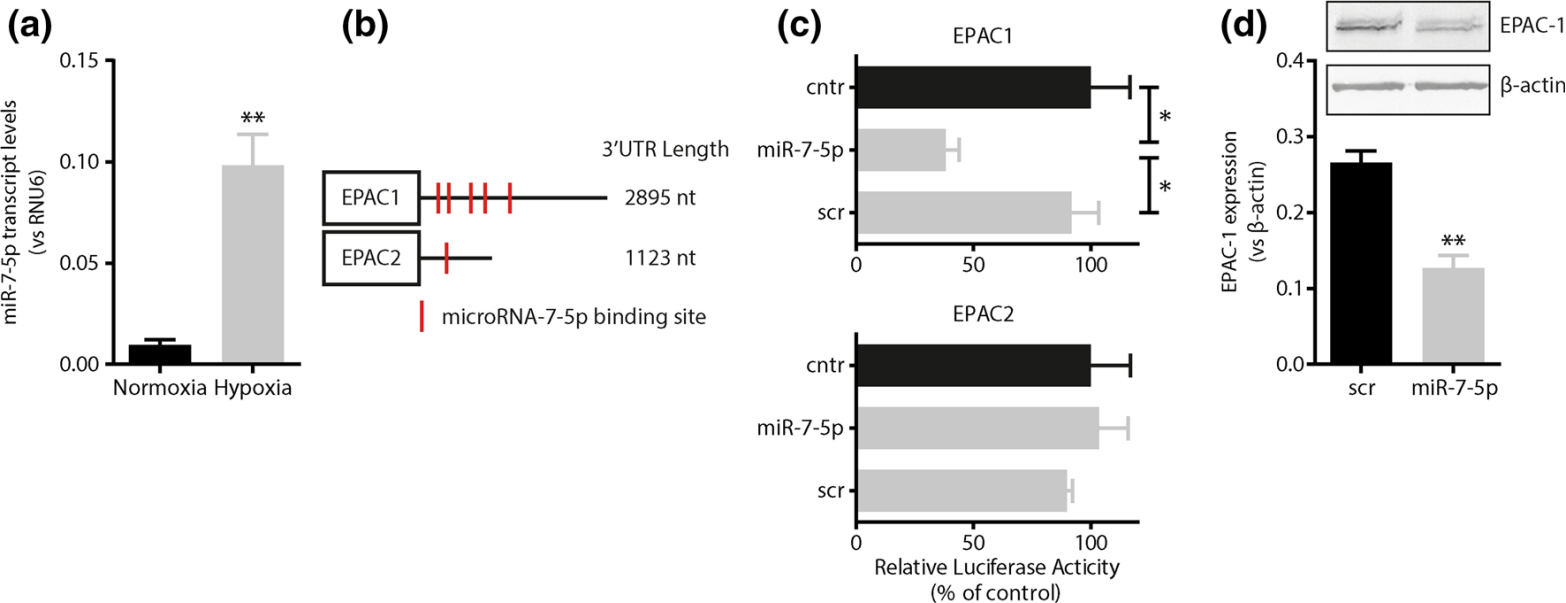
Hypoxia induces microRNA-7-mediated suppression of EPAC-1. **a** Hypoxia induces the expression of miR-7 by endothelial cells. **b** In silico analysis of the 3′UTR of EPAC-1 and EPAC-2. EPAC-1 has 5 putative miR-7 binding sites, whereas EPAC-2 has one putative miR-7 binding site. **c** Luciferase reporter assays for miR-7:3′UTR binding for EPAC-1 and EPAC-2. EPAC-1 is a genuine target of miR-7. **d** Immunoblotting of EPAC-1 in endothelial cells transfected with miR-7 mimics or scrambled sequences. MiR-7 mimics decrease EPAC-1 protein expression in cultured endothelial cells. *Cntr* control (3′UTR reporter only), *scr* scrambled sequence control; *p < 0.05. Data are expressed as mean ± SEM of at least three independent experiments

Endothelial cells exposed to 2% oxygen for 24 h increased miR-7 expression by 9.1-fold (*p* < 0.01, Fig. 2a). MicroRNA-7 contains a seed sequence that has complementarity to the 3′Untranslated Region (UTR) of EPAC-1 and EPAC-2. EPAC-1 has five putative miR-7 binding sites, whereas EPAC-2 only has one putative miR-7 binding site (Fig. 2b). To confirm that EPAC-1 and EPAC-2 are genuine targets of miR-7, we produced reporter constructs wherein the expression of luciferase is under the control of the 3′UTR of EPAC-1 or EPAC-2. Co-transfection of COS7 cells with miR-7 mimics and the EPAC-1 reporter construct decreased luciferase activity (2.6-fold) compared to scrambled controls (*p* < 0.05), whereas the luciferase activity of the EPAC-2 reporter was unaffected (Fig. 2c). These data indicate that EPAC-1 is a specific target of miR-7. Corroboratively, in endothelial cells transfected with miR-7 mimics EPAC-1 protein expression was decreased 2.1-fold (*p* < 0.05, Fig. 2d).

### cAMP signaling counteracts hypoxia-induced endothelial hyperpermeability

**Fig. 3:**
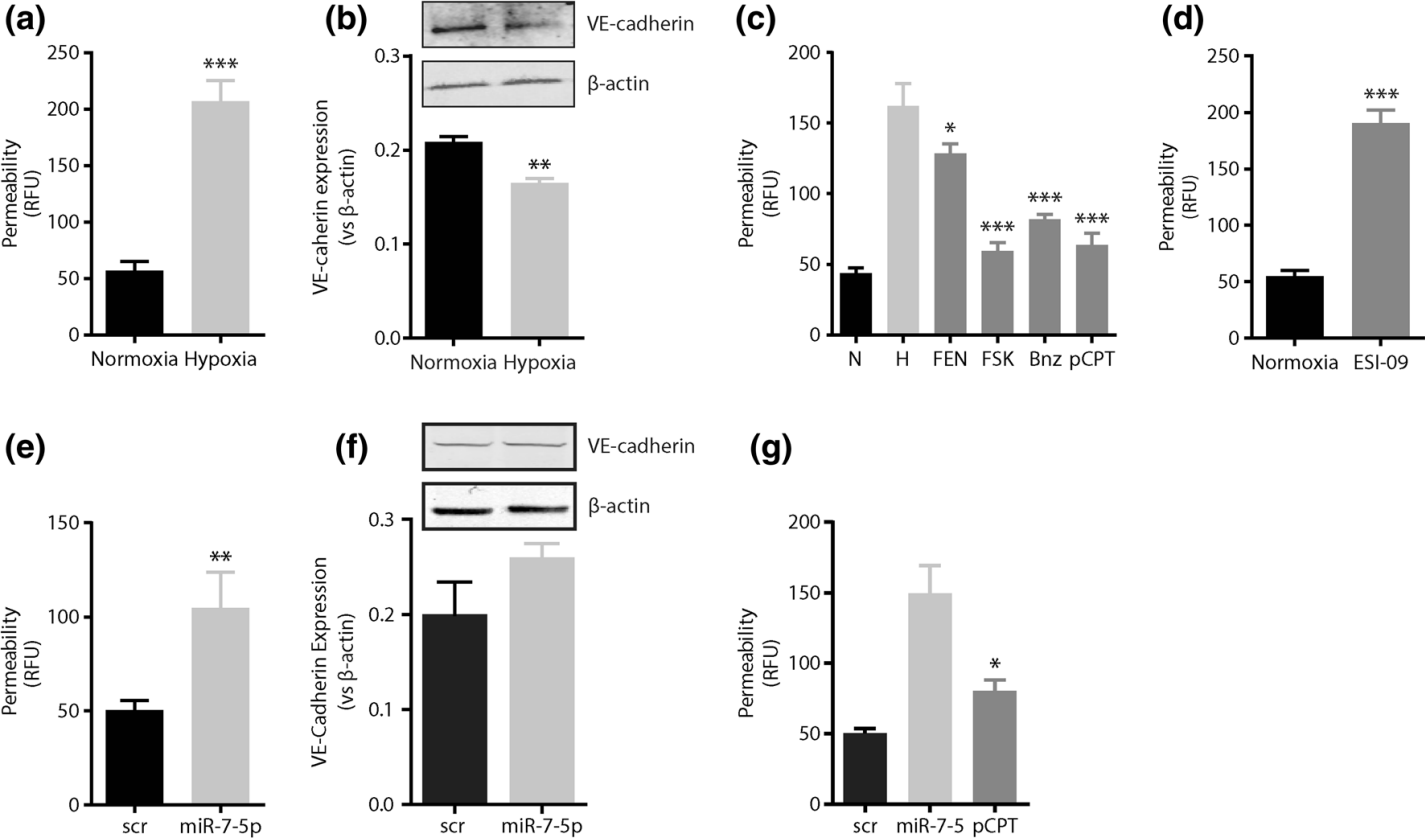
cAMP signaling counteracts hypoxia-induced endothelial hyperpermeability. **a** Permeability of endothelial monolayers grown under normoxic and hypoxic conditions. Hypoxia increases endothelial permeability. **b** Immunoblotting for VE-cadherin in endothelial cells grown under normoxic or hypoxic conditions. **c** Permeability of endothelial monolayers grown under hypoxia and stimulated with fenoterol (1 µM; FEN), forskolin (10 µM; FSK) or the cAMP analogues 6-bnz-cAMP (300 µM; PKA activator; Bnz) and 8-pCPT-2′-*O*-Me-cAMP (100 µM; EPAC-1 activator; pCPT). Stimulation of cAMP signaling decreases endothelial permeability under hypoxia. **d** Permeability of endothelial monolayers stimulated with the EPAC-1 inhibitor ESI-09 (10 µM) is increased under normoxic conditions. **e** miR-7 mimics increase endothelial monolayer permeability under normoxic conditions without affecting **f** VE-cadherin expression. **g** The miR-7-induced endothelial hyperpermeability is antagonized by the EPAC-1 activator ESI-09. *N* normoxia (20% O_2_), *H* hypoxia (2% O_2_), *Scr* scrambled sequence control; *p < 0.05; **p < 0.01; ***p < 0.001. Data are expressed as mean ± SEM of at least three independent experiments

Hypoxia causes endothelial hyperpermeability (Fig. 3a), which associates with only a minor decrease (1.2-fold, *p* < 0.01) in VE-cadherin expression (Fig. 3b). Stimulation of endothelial cells with the adenylyl cyclase activator forskolin (10 μM) significantly reduced the hypoxia-induced endothelial hyperpermeability (2.2-fold, *p* < 0.001, Fig. 3c). Similarly, the administration of the selective protein kinase A (PKA) agonist (6-Bnz-cAMP, 300 μM) and EPAC agonist (8-pCPT-2′-*O*-Me-cAMP, 100 μM), two downstream mediators of adenylyl cyclase activity, inhibited hypoxia-induced endothelial hyperpermeability [1.6-fold (*p* < 0.01) and 2.1-fold (*p* < 0.001), respectively] (Fig. 3c). Interestingly, treatment of endothelial cells with ESI-09, an antagonist to EPAC-1, induced endothelial hyperpermeability under normoxic conditions (Fig. 3d). Conversely, a β2-agonist, fenoterol (1 μM) did not reduce endothelial hyperpermeability (Fig. 3c). Also, none of the treatments altered endothelial permeability under normoxic conditions (not shown).

We next investigated if the addition of miR-7 mimics to endothelial cells would imitate the hypoxia-induced endothelial hyperpermeability. Indeed, supplementing endothelial cells with miR-7 mimics induced hyperpermeability (Fig. 3e) in the absence of hypoxia and without alterations to VE-cadherin expression (Fig. 3f). Remarkably, treating miR-7-expressing cells with 8-pCPT reduced endothelial hyperpermeability (1.7-fold, *p* < 0.05), indicating that activation of remnant EPAC-1 is sufficient to inhibit miR-7-induced endothelial hyperpermeability.

### cAMP signaling counteracts hypoxia-induced endothelial oxidative stress

**Fig. 4:**
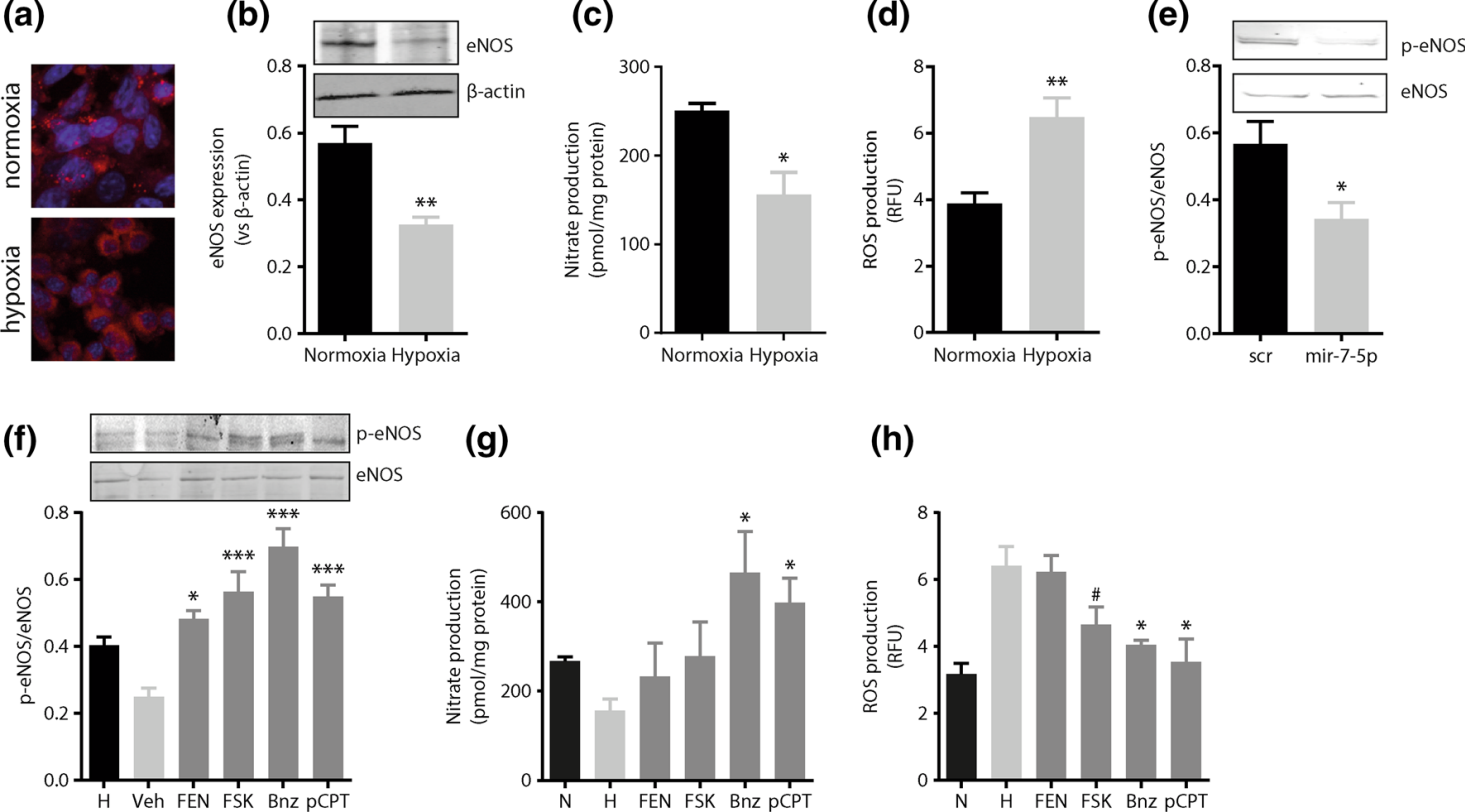
cAMP signaling counteracts hypoxia-induced endothelial oxidative stress. **a** Immunofluorescence analysis of eNOS expression by endothelial monolayers grown under normoxic and hypoxic conditions. **b** Immunoblotting for eNOS in endothelial cells grown under normoxic or hypoxic conditions. **c** Nitrite formation (indirect measurement of NO production) by endothelial cells grown under hypoxia is reduced, whereas **d** ROS production is increased. **e** miR-7 mimics decrease eNOS activity under normoxic conditions without affecting eNOS expression level. **f** Endothelial NOS phosphorylation at Ser 1177 of endothelial monolayers grown under hypoxia and stimulated with forskolin (10 µM; FSK) or the cAMP analogues 6-bnz-cAMP (300 µM; PKA activator; Bnz) and 8-pCPT-2′-*O*-Me-cAMP (100 µM; EPAC-1 activator; pCPT). Stimulation of cAMP signaling increases the phosphorylation of eNOS at Ser1177. **f** Increased eNOS phosphorylation coincides with **f** increased nitrite formation and **g** reduced ROS formation. *N* normoxia (20% O_2_), *H* hypoxia (2% O_2_); *Veh* vehicle (DMSO) control, *FEN* fenoterol (1 µM); *p < 0.05; **p < 0.01; ***p < 0.001. Data are expressed as mean ± SEM of at least three independent experiments

Endothelial NOS protein expression by cells exposed to hypoxia was reduced ~40% (*p* < 0.05, Fig. 4a, b) compared to normoxic controls, which resulted in decreased NO production (1.4-fold, *p* < 0.05, Fig. 4c) and the increased generation of reactive oxygen species (ROS) (1.6-fold, *p* < 0.01, Fig. 4d).

The addition of miR-7 mimics to endothelial cells did not cause a reduction in eNOS expression level (Fig. 4e). However, the addition of miR-7 mimics to endothelial cells did imitate the hypoxia-induced loss of eNOS activity as indicated by a reduction in eNOS phosphorylation at serine 1177 (p-eNOS/eNOS ratio; 1.7-fold decrease, *p* < 0.01; Fig. 4e).

Fenoterol (1 μM) tended to increase eNOS phosphorylation at serine 1177 (p-eNOS/eNOS ratio) in endothelial cells exposed to hypoxia (Fig. 4f), whereas forskolin (10 μM), 6-Bnz-cAMP (300 μM) and 8-pCPT-2′-*O*-Me-cAMP (100 μM) efficiently increased eNOS activation by 2.3, 2.8 and 2.2-fold respectively (all *p* < 0.05, Fig. 4f). Corroboratively, NO production in hypoxia-treated endothelial cells was increased (1.4- to 1.6-fold) by these activators of cAMP signaling (Fig. 4g), and ROS production was decreased to a similar extend (Fig. 4h).

### EPAC-1 and microRNA-7 alterations in diabetic retinopathy

**Fig. 5:**
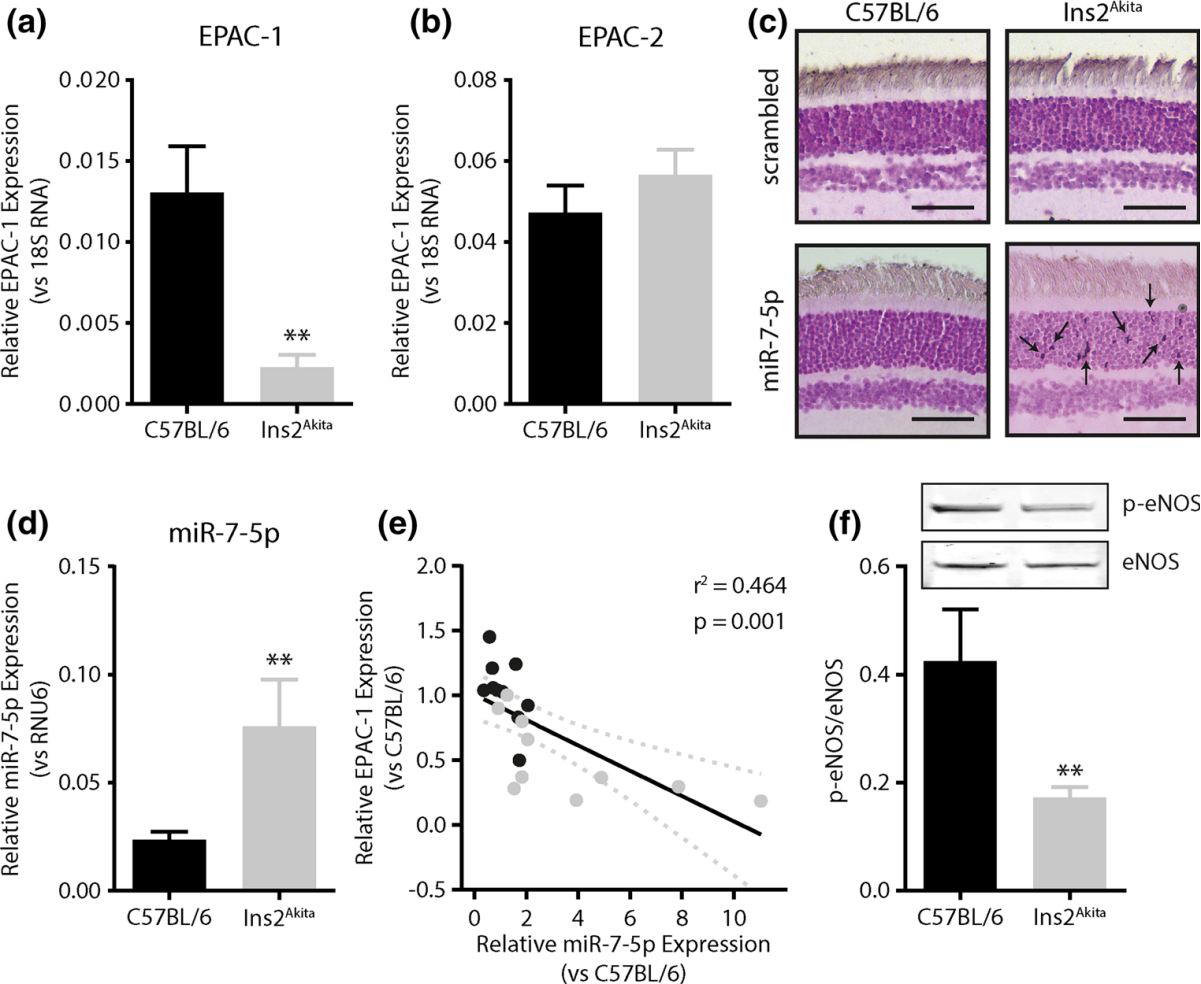
EPAC-1 and microRNA-7 alterations in diabetic retinopathy. **a** EPAC-1 gene expression in retinal lysates of 6-month-old spontaneous diabetic heterozygous Ins2Akita^+/−^ and control non-diabetic littermates (homozygous Ins2Akita^−/−^ mice). **b** EPAC-1 gene expression in retinal lysates of 6-month-old spontaneous diabetic heterozygous Ins2Akita^+/−^ and control non-diabetic littermates (homozygous Ins2Akita^−/−^ mice). **c** In situ hybridization using scrambled probes or miR-7-5p-specific probes on retinal digests from 6-month-old spontaneous diabetic heterozygous Ins2Akita^+/−^ and control non-diabetic littermates. **d** MicroRNA-7-5p expression in retinal lysates of spontaneous diabetic Ins2Akita^+/−^ and control mice. **e** Association between the EPAC-1 and miRNA-7-5p expression levels in non-diabetic and diabetic Ins2Akita mice. **f** Levels of phosph-eNOS (Ser1177) and eNOS in non-diabetic and diabetic Ins2Akita mice

In the Ins2Akita model for diabetic retinopathy (Table 1), retinal EPAC-1 levels were reduced (~5.7-fold, *p* < 0.01) compared to non-diabetic control mice (Fig. 5a), whereas gene expression levels of EPAC-2 remained unchanged (Fig. 5b). MiR-7-5p was detected by in situ hybridization in the retinae from diabetic Ins2Akita mice (Fig. 5c), where its expression of miR-7 was increased (3.2-fold, *p* < 0.01, Fig. 5d) compared to non-diabetic controls. In control C57BL/6 mice, miR-7-5p levels remained below the detection limit for in situ hybridization (Fig. 5c). EPAC-1 expression levels associated with miR-7-5p expression levels to a high extend (*r* ^2^ = 0.464, *p* < 0.001, Fig. 5c) indicating that the hypoxia-induced expression of miR-7 might underlie the loss of EPAC-1 expression in diabetic retinopathy. In the Ins2Akita mice, endothelial hyperpermeability, as assessed by leakage of fluorescently labeled low molecular weight dextran, was not observed (data not shown). However, eNOS activation decreased 2.4-fold (*p* < 0.01, Fig. 5f) compared to control mice, suggestive of reduced EPAC signaling.

**Table 1:** Metabolic data of Diabetic Ins2^Akita^ mice

	C57BL/6	Ins2^Akita^	*p* value
Age (months)	6	6	
Body weight (g)	34.65 ± 3.69	26.24 ± 1.48	<0.0001
Blood glucose (mg/dl)	209 ± 33	>600	<0.0001
HB1Ac (%)	6.38 ± 0.68	13.33 ± 1.03	0.0005

Besides EPAC-1 [21], miR-7 targets a number of additional gene transcripts (Suppl. Table 1). We analyzed the expression of 23 reported miR-7-5p target genes in endothelial cells that express miR-7-5p (Suppl. Table 2) and in retinal isolations from diabetic Ins2Akita mice (Suppl. Table 4) and found no other transcript which was decreased in both conditions.

## Discussion

Here, we show that hypoxia induces the expression of miR-7 by endothelial cells in vitro and in vivo, reducing the expression of EPAC-1. The hypoxia-induced reduction in EPAC-1 levels results in endothelial hyperpermeability and a NO/ROS imbalance and is associated with the development of (diabetic) retinopathy. Activation of EPAC-1 by forskolin or the cAMP analogue 8-pCPT reduces the sensitivity of EPAC-1 to oxidative stress and restores the endothelial permeability barrier and rescues NO production by eNOS. These data suggest that EPAC-1 is an appropriate drug target for the treatment of endothelial dysfunction during (diabetic) retinopathy.

(Diabetic) Retinopathy is characterized by hypoxia-induced vascular dysfunction, resulting in the degradation of the blood-retinal barrier (BRB) and concomitant macular edema formation. Herein, hypoxia induces the loss of endothelial cell–cell junctions and oxidative stress [24, 25]. Although cAMP signaling is commonly known to regulate endothelial permeability and NO production [26, 27], little is known on the role of cAMP signaling in retinopathy. Yet, agonist to the β-adrenergic system [28, 29] and taurine [30] prevent retinal endothelial hyperpermeability in part through the activation of cAMP signaling.

Considering these antecedents, we investigated cAMP signaling in the oxygen-induced retinopathy model [22] and found a marked decrease in the expression of the cAMP signaling intermediate EPAC-1. Concurrently, exposing endothelial cells to a hypoxia challenge in vitro similarly reduced EPAC-1 expression levels, suggesting that the loss of EPAC-1 might contribute to the hypoxia-induced retinopathy. Indeed, adenosine reduces inflammatory retinopathy by activating EPAC1-1 [31].

We had previously found an association between EPAC-1 and miR-7 expression levels in airway smooth muscle cells [21]. MicroRNAs are endogenous translational repressors of gene expression. Hence, we investigated if hypoxia could affect EPAC-1 expression through miR-7. Indeed, hypoxia induced the expression of miR-7 in endothelial cells and transfection of endothelial cells with miR-7 mimics reduced EPAC-1 expression by ~50%.

The integrity of the BRB is highly dependent on endothelial adherence junctions that consist of VE-cadherin and associated catenins. Hypoxia reduces the presence of VE-cadherin in the endothelial cell–cell junctions [32], resulting in endothelial hyperpermeability. This change in permeability might be derived from the hypoxia-induced reduction in EPAC-1 expression, as EPAC-1 is pivotal in maintaining VE-cadherin at the cell–cell junction through the activation of Rac [10, 26]. Corroboratively, inhibition of EPAC-1 activity with the small molecule ESI-09 or miR-7-5p mimics increases the endothelial permeability. Interestingly, hypoxia-mediated endothelial hyperpermeability was associated with a decreased in VE-cadherin expression, whereas hyperpermeability induced by miR-7-5p mimics was not. As both models are characterized by a similar reduction in EPAC-1, these data suggest that hypoxia-driven hyperpermeability involves at least one other cascade that results in the degradation of VE-cadherin. Activation of the remnant EPAC-1 by forskolin or the cAMP analogue 8-pCPT antagonizes the hypoxia or miR-7-induced endothelial hyperpermeability. These data are corroborated by earlier findings of Aslam et al. [32], who describe the restoration of VE-cadherin at the cell–cell junction by 8-pCPT under hypoxic conditions.

As our data implies that miR-7-5p underlies hyperpermeability in oxygen-induced retinopathy and eNOS uncoupling in diabetic retinopathy, inhibition of miR-7-5p, or EPAC-1 activation seem a promising approach to restore retinopathy. Unfortunately, the current methodology to assess permeability in vivo, i.e., Evans Blue dye leakage, or FITC-Dextran leakage, is too insensitive to provide reproducible results on the relative permeability in Ins2Akita. The development of novel technologies, such as scanning laser ophthalmoscopy or optical coherence tomography [33] may provide better resolution in the near future. Alternatively, the streptozotocin-induced rat model for retinopathy, which produced a high retinal hyperpermeability, could be used to investigate the efficacy of EPAC activating drugs.

Additionally, it would be of interest to investigate if EPAC-1 activation can rescue the VEGFa-induced endothelial hyperpermeability, as this is not only associated to (diabetic) retinopathy, but also with endothelial hyperpermeability in tumors.

Besides endothelial hyperpermeability, in retinopathy, hypoxia contributes to endothelial oxidative stress orchestrated by eNOS [34]. Here, we found a marked reduction in eNOS expression and phosphorylation during hypoxia, which coincided with decreased NO levels and elevated ROS production. Interestingly, the reduction in eNOS mRNA stability and eNOS activity are dependent on the activity of Rho-kinase [35]. It is conceivable that the hypoxia-induced suppression of EPAC-1 expression and activity would concomitantly increase Rho-kinase activity, which would in turn cause the observed reduction in eNOS expression and activation. Besides, the observed reduction in eNOS phosphorylation at Ser1177 might result in eNOS uncoupling and the concomitant generation of ROS. Indeed, in the present study we observe decreased eNOS activity under hypoxic conditions, reflected by reduced nitrite formation, and increased ROS production. Moreover, endothelial cells that were transfected with miR-7-5p mimics showed decreased eNOS activity and eNOS activity is decreased in the retinae of diabetic Ins2Akita mice. Activation of EPAC-1 in endothelial cells that received a hypoxic challenge using forkolin or 8-pCPT completely rescued this phenotype and restored NO production and inhibited the formation of ROS.

A potential limitation of our study is the use of umbilical vein endothelial cells, which are macrovascular cells. Although we cannot fully exclude that retinal microvasculature endothelial cells would behave different with respect to miR-7-5p or EPAC-1 stimulation, in preliminary experiments we have found no difference between the umbilical vein endothelial cells and dermal microvascular endothelial cells (data not shown).

**Fig. 6:**
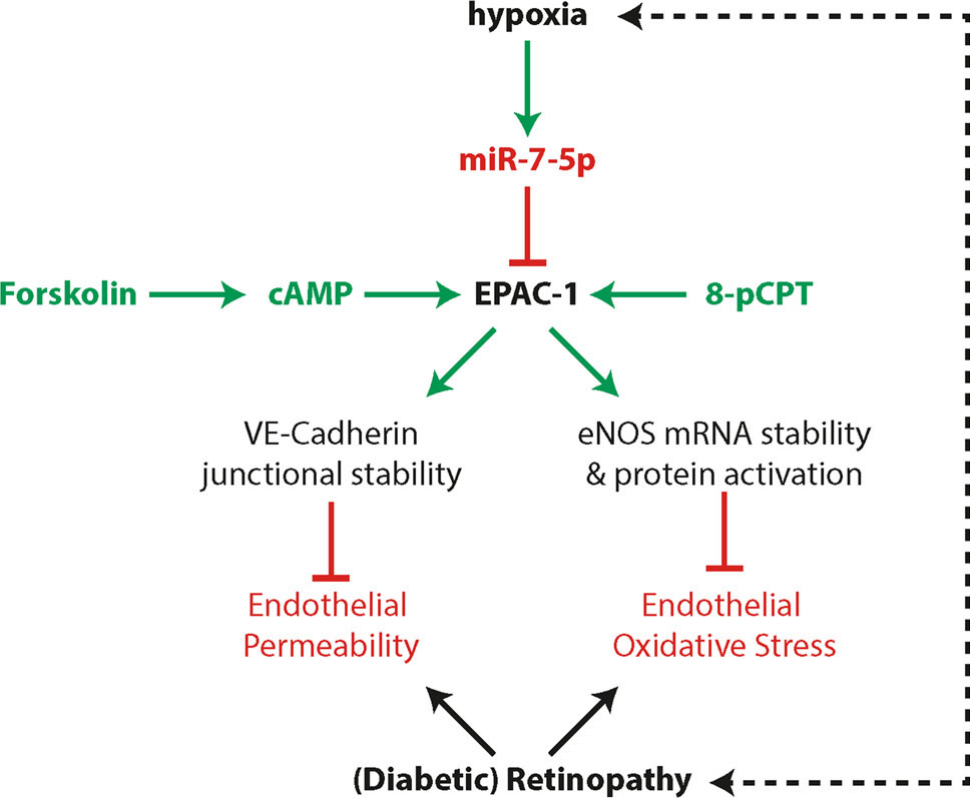
Hypoxia-mediated repression of EPAC-1 by microRNA-7 in retinopathy. Schematic representation of the study outcomes. Hypoxia during retinopathy increases the expression of microRNA-7, which in turn reduces the protein availability of EPAC-1. The loss of EPAC-1 in endothelial cells causes endothelial junctional instability and concurrently hyperpermeability, as well as the loss of eNOS expression and eNOS activity, resulting in oxidative stress. Combined, hyperpermeability and oxidative stress might further reduce the oxygen transport creating a feed-forward mechanism that aggravates retinopathy. Stimulators of cAMP signaling and EPAC-1 efficiently activate the remnant EPAC-1 protein, which antagonizes the hypoxia-induced damage. Therefore, EPAC-1 is an appropriate drugable target for the treatment of endothelial dysfunction during (diabetic) retinopathy

In summary (Fig. 6), here we report that the hypoxia-induced reduction in EPAC-1 expression and activity contributes to the generation of retinopathy through the disruption of the endothelial barrier in the OIR model, or by eNOS uncoupling (disturbing the NO/ROS balance) in the Ins2Akita model. The reduction in EPAC-1 expression is in part due to the hypoxia-induced expression of miR-7. Pharmacological activation of EPAC-1 by forskolin or 8-pCPT antagonizes the hypoxia-induced endothelial dysfunction. Therefore, EPAC-1 resembles an efficacious and druggable target molecule for the amelioration of (diabetic) retinopathy.

## Electronic supplementary material

Below is the link to the electronic supplementary material.

Supplementary material 1 (DOCX 32 kb)

## Abbreviations

cAMP: 3′,5′-Cyclic adenosine monophosphate
eNOS: Endothelial nitric oxide synthase
EPAC: Exchange protein activated by cAMP
GEFs: Guanine nucleotide exchange factors
miR: MicroRNA
ROS: Reactive oxygen species

## References

[1] Grimm C, Willmann G (2012). Hypoxia in the eye: a two-sided coin. High Alt Med Biol.

[2] Pearlstein DP, Ali MH, Mungai PT, Hynes KL, Gewertz BL, Schumacker PT (2002). Role of mitochondrial oxidant generation in endothelial cell responses to hypoxia. Arterioscler Thromb Vasc Biol.

[3] Yan SF, Ogawa S, Stern DM, Pinsky DJ (1997). Hypoxia-induced modulation of endothelial cell properties: regulation of barrier function and expression of interleukin-6. Kidney Int.

[4] Koto T, Takubo K, Ishida S (2007). Hypoxia disrupts the barrier function of neural blood vessels through changes in the expression of claudin-5 in endothelial cells. Am J Pathol.

[5] Ten VS, Pinsky DJ (2002). Endothelial response to hypoxia: physiologic adaptation and pathologic dysfunction. Curr Opin Crit Care.

[6] Pinsky DJ, Yan SF, Lawson C (1995). Hypoxia and modification of the endothelium: implications for regulation of vascular homeostatic properties. Semin Cell Biol.

[7] Han JA, Seo EY, Kim HJ (2013). Hypoxia-augmented constriction of deep femoral artery mediated by inhibition of eNOS in smooth muscle. Am J Physiol Cell Physiol.

[8] De Rooij J, Zwartkruis FJ, Verheijen MH (1998). EPAC is a Rap1 guanine-nucleotide-exchange factor directly activated by cyclic AMP. Nature.

[9] Breckler M, Berthouze M, Laurent AC, Crozatier B, Morel E, Lezoualc’h F (2011). Rap-linked cAMP signaling EPAC proteins: compartmentation, functioning and disease implications. Cell Signal.

[10] Kooistra MR, Corada M, Dejana E, Bos JL (2005). EPAC1 regulates integrity of endothelial cell junctions through VE-cadherin. FEBS Lett.

[11] Birukova AA, Tian Y, Dubrovskyi O (2012). VE-cadherin *trans*-interactions modulate Rac activation and enhancement of lung endothelial barrier by iloprost. J Cell Physiol.

[12] Cullere X, Shaw SK, Andersson L, Hirahashi J, Luscinskas FW, Mayadas TN (2005). Regulation of vascular endothelial barrier function by EPAC, a cAMP-activated exchange factor for Rap GTPase. Blood.

[13] Sehrawat S, Cullere X, Patel S, Italiano J, Mayadas TN (2008). Role of EPAC1, an exchange factor for Rap GTPases, in endothelial microtubule dynamics and barrier function. Mol Biol Cell.

[14] Zieba BJ, Artamonov MV, Jin L (2011). The cAMP-responsive Rap1 guanine nucleotide exchange factor, EPAC, induces smooth muscle relaxation by down-regulation of RhoA activity. J Biol Chem.

[15] Roberts OL, Kamishima T, Barrett-Jolley R, Quayle JM, Dart C (2013). Exchange protein activated by cAMP (EPAC) induces vascular relaxation by activating Ca2+-sensitive K+ channels in rat mesenteric artery. J Physiol.

[16] Rampersad S, Hubert F, Umana M (2015). cAMP-signaling via EPAC1 mediates vascular endothelial cell adaptation to fluid-shear stress. FASEB J.

[17] Garcia-Morales V, Cuinas A, Elies J, Campos-Toimil M (2014). PKA and EPAC activation mediates cAMP-induced vasorelaxation by increasing endothelial no production. Vascul Pharmacol.

[18] Birukova AA, Burdette D, Moldobaeva N, Xing J, Fu P, Birukov KG (2010). Rac GTPase is a hub for protein kinase a and EPAC signaling in endothelial barrier protection by cAMP. Microvasc Res.

[19] Birukova AA, Zagranichnaya T, Alekseeva E, Bokoch GM, Birukov KG (2008). EPAC/Rap and PKA are novel mechanisms of ANP-induced Rac-mediated pulmonary endothelial barrier protection. J Cell Physiol.

[20] Schmidt M, Dekker FJ, Maarsingh H (2013). Exchange protein directly activated by cAMP (EPAC): a multidomain camp mediator in the regulation of diverse biological functions. Pharmacol Rev.

[21] Oldenburger A, Van Basten B, Kooistra W (2014). Interaction between EPAC1 and Mirna-7 in airway smooth muscle cells. Naunyn Schmiedebergs Arch Pharmacol.

[22] Smith LE, Wesolowski E, Mclellan A (1994). Oxygen-induced retinopathy in the mouse. Invest Ophthalmol Vis Sci.

[23] Hammes HP, Brownlee M, Jonczyk A, Sutter A, Preissner KT (1996). Subcutaneous injection of a cyclic peptide antagonist of vitronectin receptor-type integrins inhibits retinal neovascularization. Nat Med.

[24] Karimova A, Pinsky DJ (2001). The endothelial response to oxygen deprivation: biology and clinical implications. Intensive Care Med.

[25] Kaur C, Foulds WS, Ling EA (2008). Blood-retinal barrier in hypoxic ischaemic conditions: basic concepts, clinical features and management. Prog Retin Eye Res.

[26] Fukuhara S, Sakurai A, Sano H (2005). Cyclic AMP potentiates vascular endothelial cadherin-mediated cell–cell contact to enhance endothelial barrier function through an EPAC-Rap1 signaling pathway. Mol Cell Biol.

[27] Bae SW, Kim HS, Cha YN, Park YS, Jo SA, Jo I (2003). Rapid increase in endothelial nitric oxide production by bradykinin is mediated by protein kinase A signaling pathway. Biochem Biophys Res Commun.

[28] Zink S, Rosen P, Lemoine H (1995). Micro- and macrovascular endothelial cells in beta-adrenergic regulation of transendothelial permeability. Am J Physiol.

[29] Jiang Y, Zhang Q, Liu L, Tang J, Kern TS, Steinle JJ (2013). Beta2-adrenergic receptor knockout mice exhibit A diabetic retinopathy phenotype. PLoS ONE.

[30] Pavan B, Capuzzo A, Forlani G (2014). High glucose-induced barrier impairment of human retinal pigment epithelium is ameliorated by treatment with Goji berry extracts through modulation of cAMP levels. Exp Eye Res.

[31] Ibrahim AS, El-Shishtawy MM, Zhang W, Caldwell RB, Liou GI (2011). A 2A adenosine receptor (A 2A AR) as a therapeutic target in diabetic retinopathy. Am J Pathol.

[32] Aslam M, Schluter K-D, Rohrbach S (2013). Hypoxia–reoxygenation-induced endothelial barrier failure: role of RhoA, Rac1 and myosin light chain kinase. J Physiol.

[33] Mclenachan S, Magno AL, Ramos D (2015). Angiography reveals novel features of the retinal vasculature in healthy and diabetic mice. Exp Eye Res.

[34] Caldwell RB, Zhang W, Romero MJ, Caldwell RW (2010). Vascular dysfunction in retinopathy—an emerging role for arginase. Brain Res Bull.

[35] Takemoto M, Sun J, Hiroki J, Shimokawa H, Liao JK (2002). Rho-kinase mediates hypoxia-induced downregulation of endothelial nitric oxide synthase. Circulation.

