# Supplementary material for: The microRNA-7-mediated reduction in EPAC-1 contributes to vascular endothelial permeability and eNOS uncoupling in murine experimental retinopathy
Source: Acta Diabetol. 2017 Mar 28;54(6):581–91. doi: 10.1007/s00592-017-0985-y (PMC5429352; doi:10.1007/s00592-017-0985-y)
Supplement: Supplementary file 1 — Supplementary material 1 (DOCX 32 kb) [file 592_2017_985_MOESM1_ESM.docx]

**Supplemental table 1. Primers used to analyze human microRNA-7 target genes.**

| **Gene** | **Protein name** | **Ref Sequence** | **Sense Primer** | **Antisense Primer** |
| --- | --- | --- | --- | --- |
| **EPAC-1**  **(RAPGEF3)** | Exchange protein directly activated by cAMP-1 | NM_001098531.2 | CTGCTGAGGGAGCAGTGG | AGCCAAACAGGCAAGTTCC |
| **EPAC-2**  **(RAPGEF4)** | Exchange protein directly activated by cAMP-2 | NM_007023.3 | GCACCTCACATGATAAGAGATAGAAA | CCGGGAGTGAACACATGG |
|  |  |  |  |  |
| **ACK1 (TNK2)** | Activated CDC42 kinase 1 | NM_001791.3 | GCTCCATGGGGGAGAGAT | CTCCTCTGGCTGCATTCTG |
| **CDK1** | Cyclin-dependent kinase 1 | NM_001786.4 | TGGATCTGAAGAAATACTTGGATTCTA | CAATCCCCTGTAGGATTTGG |
| **CKS2** | CDC28 protein kinase regulatory subunit 2 | NM_001827.2 | TTCGACGAACACTACGAGTACC | AGCCTAGACTCTGTTGGACACC |
| **EGFR** | Epidermal growth factor receptor | NM_005228.3 | TTCCTCCCAGTGCCTGAA | GGTTCAGAGGCTGATTGTGAT |
| **EIF4E** | Eukaryotic translation initiation factor 4E | NM_001968.3 | CAATCCGGTTTGAATCTCATTT | TTAGGAGTAGGGGTGGTTTCC |
| **GDF5** | Growth differentiation factor 5 | NM_001319138.1 | GCTTTATTGACAAAGGGCAAGA | GGGCACTAATGTCAAACACG |
| **IGF1R** | Insulin-like growth factor 1 receptor | NM_000875.4 | TTCAGCGCTGCTGATGTG | AAGTTCCCGGCTCATGGT |
| **IRS2** | Insulin receptor substrate 2 | NM_003749.2 | TTCTTGTCCCACCACTTGAA | CTGACATGTGACATCCTGGTG |
| **KLF4** | Kruppel-like factor 4 | NM_001314052.1 | GGGAGAAGACACTGCGTCA | GGAAGCACTGGGGGAAGT |
| **MAPKAP1** | Mitogen-activated protein kinase associated protein 1 | NM_001006617.1 | CAGAGCGAGCCCAATGTC | CTCAAAAACCCCGTCTGC |
| **MKNK1** | MAP kinase interacting serine/threonine kinase 1 | NM_003684.5 | CAAGCTCCAGAAAAGGGACTC | TCAGGTCCATTGTGCTGCT |
| **MKNK2** | MAP kinase interacting serine/threonine kinase 2 | NM_017572.3 | GCAGACCTGCATCAACCTG | CTGGCTGCTTCTCAATGATCT |
| **MTOR** | Mechanistic target of rapamycin | NM_004958.3 | ACCCAACATCTCTTCGGAAA | TTGGTCGATAAAGGGATGACA |
| **NR4A3** | Nuclear receptor subfamily 4 group A member 3 | NM_006981.3 | TCTCAGTGTTGGAATGGTAAAAGA | GGTTTGGAAGGCAGACGAC |
| **RPS6KB1 (P70S6K)** | 70 kDa Ribosomal protein S6 kinase B1 | NM_001272060.1 | TAAAGGGGGCTATGGAAAGG | TTAAGCACCTTCATGGCAAAT |
| **PAK1** | p21 (RAC1) activated kinase 1 | NM_001128620.1 | CACGGTTTGAGAAGATTGGAC | GGGCTGCTGCTGAAGATT |
| **PAX6** | Paired box 6 | NM_000280.4 | CAGCTTCACCATGGCAAATA | GGCAGCATGCAGGAGTATG |
| **PIK3CD** | phosphatidylinositol-4,5-bisphosphate 3-kinase catalytic subunit delta | NM_005026.3 | CGGGATGAGCAGAGCAAC | ACCACAGGGACACAGAGGA |
| **RAF1** | Raf-1 proto-oncogene, serine/threonine kinase | NM_002880.3 | AGAGCGGGCACCAGTATCT | CACTGGCTTCTATTTCCCAATAA |
| **PSME3 (REGγ)** | Proteasome activator subunit 3 | NM_005789.3 | AGCCTTCGGCTCATCATATC | TTCAGGATCATGTCATGTAGAGTG |
| **RELA** | RELA proto-oncogene, NF-kB subunit | NM_021975.3 | CGGGATGGCTTCTATGAGG | CTCCAGGTCCCGCTTCTT |
| **SGK-1** | Serum/glucocorticoid regulated kinase 1 | NM_005627.3 | TCCTAGACTACATTAATGGTGGAGAGT | ATAGAAACGAGCCCGTGGTT |
| **SMARCD1** | SWI/SNF related, matrix associated, actin dependent regulator of chromatin, subfamily d, member 1 | NM_003076.4 | TGGGAGCTTCGGGTAGAAG | TGGCATCATATTTGGACAAGG |
| **SNCA** | Synuclein, alpha | NM_000345.3 | GAGGGAGTGGTGCATGGT | TGCTGTCACACCCGTCAC |

**Supplementary Table 2. Expression of microRNA-7 target genes in human endothelial cells.**

| **Gene** | **Ct miR-7  (n=5)** | **Ct SCR Control  (n=5)** | **Fold Change (miR-7 vs SCR)** | **P-value** | **Reference to target identification** |
| --- | --- | --- | --- | --- | --- |
| **EPAC-1 (RAPGEF3)** | **27.67±0.08** | **26.11±0.16** | **0.34±0.02** | **0.001** | **[1]** |
| **EPAC-2 (RAPGEF4)** | 25.84±0.06 | 25.88±0.09 | 1.05±0.05 | 0.266 |  |
|  |  |  |  |  |  |
| **ACK1 (TNK2)** | 29.78±0.08 | 29.73±0.13 | 0.99±0.05 | 0.434 | [2] |
| **CDK1** | **22.48±0.09** | **22.21±0.15** | **0.84±0.04** | **0.026** | **[3]** |
| **CKS2** | 22.42±0.03 | 22.31±0.17 | 0.94±0.03 | 0.110 | [4] |
| **EGFR** | **22.97±0.09** | **25.33±0.72** | **3.65±0.31** | **0.002** | **[3,5,6]** |
| **EIF4E** | 29.25±0.04 | 30.04±0.55 | 1.43±0.03 | 0.076 | [3,7] |
| **GDF5** | 29.85±0.07 | 29.70±0.14 | 0.92±0.05 | 0.088 | [8] |
| **IGF1R** | 23.08±0.14 | 22.57±0.12 | 0.69±0.08 | 0.093 | [9] |
| **IRS2** | 27.90±0.04 | 27.76±0.17 | 0.92±0.04 | 0.118 | [10] |
| **KLF4** | 26.94±0.05 | 26.76±0.12 | 0.90±0.04 | 0.076 | [11,12] |
| **MAPKAP1** | 22.44±0.06 | 22.42±0.13 | 1.00±0.04 | 0.466 | [7] |
| **MKNK1** | 24.74±0.11 | 25.04±0.10 | 1.25±0.08 | 0.051 | [7] |
| **MKNK2** | 23.27±0.05 | 23.21±0.14 | 0.92±0.05 | 0.303 | [7] |
| **MTOR** | 23.34±0.05 | 23.36±0.11 | 0.98±0.03 | 0.427 | [3,13,14] |
| **NR4A3** | 31.63±0.14 | 31.61±0.36 | 0.85±0.06 | 0.308 | [15] |
| **RPS6KB1 (P70S6K)** | 22.81±0.05 | 23.04±0.19 | 1.10±0.03 | 0.322 | [7,13] |
| **PAK1** | 25.45±0.05 | 25.58±0.13 | 1.05±0.02 | 0.345 | [16,17] |
| **PAX6** | ND | ND | ND | ND | [18] |
| **PIK3CD** | 26.35±0.10 | 26.25±0.13 | 0.91±0.04 | 0.216 | [13] |
| **RAF1** | **32.40±0.27** | **33.24±0.11** | **1.84±0.29** | **0.016** | **[3,6,19]** |
| **PSME3 (REGγ)** | 22.32±0.28 | 23.36±0.60 | 1.61±0.28 | 0.122 | [20] |
| **RELA** | 21.49±0.05 | 21.44±0.10 | 0.94±0.04 | 0.296 | [21,22] |
| **SGK1** | 20.74±0.02 | 20.79±0.12 | 0.99±0.04 | 0.485 | [14] |
| **SMARCD1** | **24.83±0.23** | **23.24±0.34** | **0.32±0.06** | **0.012** | **[23]** |
| **SNCA** | 21.69±0.14 | 21.75±0.14 | 1.02±0.11 | 0.451 | [24-26] |

**Supplemental table 3. Primers used to analyze murine microRNA-7 target genes.**

| **Gene** | **Protein name** | **Ref Sequence** | **Sense Primer** | **Antisense Primer** |
| --- | --- | --- | --- | --- |
| **EPAC-1**  **(RAPGEF3)** | Exchange protein directly activated by cAMP-1 | NM_001177810.1 | Gggaacgtatctcctcagacc | ggactttgtccccaactcg |
| **EPAC-2**  **(RAPGEF4)** | Exchange protein directly activated by cAMP-2 | NM_001204165.1 | Tggaaccaactggtatgctg | cccagagtgcagatggtca |
|  |  |  |  |  |
| **ACK1 (TNK2)** | Activated CDC42 kinase 1 | NM_016788.3 | ttcgactggaacctagagcaa | cagtcaaccatcagcgtttg |
| **CDK1** | Cyclin-dependent kinase 1 | NM_007659.3 | agaacttcgacatccaaatatagtca | ccatggacaggaactcaaaga |
| **CKS2** | CDC28 protein kinase regulatory subunit 2 | NM_025415.3 | cccaaaactcatctgatgtcc | taaagagaagaatatgcggttctg |
| **EGFR** | Epidermal growth factor receptor | NM_207655.2 | ttggaatcaattttacaccgaat | gttcccacacagtgacacca |
| **EIF4E** | Eukaryotic translation initiation factor 4E | NM_007917.4 | tgggctctatacaaccatatcca | ttcccacataggctcaatcc |
| **GDF5** | Growth differentiation factor 5 | NM_008109.2 | tcctaagctctttaagggagagc | aagtcaccaggcacaaaggt |
| **IGF1R** | Insulin-like growth factor 1 receptor | NM_010513.2 | atcagaaagtacgccgatgg | cacttctgtcttgggattttcc |
| **IRS2** | Insulin receptor substrate 2 | NM_001081212.1 | ttccccttcctccttacagc | aggcaggcggtataggtctc |
| **KLF4** | Kruppel-like factor 4 | NM_010637.3 | cgggaagggagaagacact | gagttcctcacgccaacg |
| **MAPKAP1** | Mitogen-activated protein kinase associated protein 1 | NM_177345.4 | caggcaagcagtccatattgt | ttgcagttgtacccacatgac |
| **MKNK1** | MAP kinase interacting serine/threonine kinase 1 | NM_001285487.1 | cgagaagccagtagagtggtg | acggtgagcaatgcctttag |
| **MKNK2** | MAP kinase interacting serine/threonine kinase 2 | NM_021462.4 | aggccagcgtggtagtacag | tctggctttaggtccctgtg |
| **MTOR** | Mechanistic target of rapamycin | NM_020009.2 | acgtgttcagacggatctca | cagaagtttgggggacacat |
| **NR4A3** | Nuclear receptor subfamily 4 group A member 3 | NM_015743.3 | gcccagcttcagtaccttca | gcatttggtacaggcaggag |
| **RPS6KB1 (P70S6K)** | 70 kDa Ribosomal protein S6 kinase B1 | NM_001114334.1 | gctggagaagtccaagctca | ggcttaaagggaggttccac |
| **PAK1** | p21 (RAC1) activated kinase 1 | NM_011035.2 | acggttcgagaagattggac | tgtggctacatccattgcag |
| **PAX6** | Paired box 6 | NM_001244198.2 | caccagactcacctgacacc | accgcccttggttaaagtc |
| **PIK3CD** | phosphatidylinositol-4,5-bisphosphate 3-kinase catalytic subunit delta | NM_001164052.1 | gctctggagaagatcctggag | gtccttctcatgttcgtacagttc |
| **RAF1** | Raf-1 proto-oncogene, serine/threonine kinase | NM_029780.3 | caggatgattgaggatgcaa | agcctgttggactcaggttg |
| **PSME3 (REGγ)** | Proteasome activator subunit 3 | NM_011192.3 | ccgcactgtcacagagattg | ggagagtgacatactgattcctca |
| **RELA** | RELA proto-oncogene, NF-kB subunit | NM_009045.4 | tgcccagaccgcagtatc | ggattcgctggctaatgg |
| **SGK-1** | Serum/glucocorticoid regulated kinase 1 | NM_001161845.2 | tttccaaagggggatgct | tgttggcatgattacattgttct |
| **SMARCD1** | SWI/SNF related, matrix associated, actin dependent regulator of chromatin, subfamily d, member 1 | NM_031842.2 | gggagctccgggtagaag | tggcgtcatatttggacaag |
| **SNCA** | Synuclein, alpha | NM_001042451.2 | tggcagtgaggcttatgaaa | gcttcaggctcatagtcttgg |

**Supplementary Table 4. Expression of microRNA-7 target genes in murine experimental retinopathy.**

| **Gene** | **Ct Diabetic  (n=5)** | **Ct Control  (n=5)** | **Fold Change (DC vs Cntr)** | **P-value** | **Reference to target identification** |
| --- | --- | --- | --- | --- | --- |
| **EPAC-1 (RAPGEF3)** | **32.60±0.98** | **29.41±1.29** | **0.18±0.06** | **0.011** | **[1]** |
| **EPAC-2 (RAPGEF4)** | 27.79±0.79 | 27.50±1.34 | 1.20±0.13 | 0.354 |  |
|  |  |  |  |  |  |
| **ACK1 (TNK2)** | **25.83±1.46** | **25.85±0.94** | **1.35±0.12** | **0.048** | **[2]** |
| **CDK1** | 34.57±0.95 | 34.37±1.55 | 1.06±0.15 | 0.778 | [3] |
| **CKS2** | 24.81±1.23 | 24.43±1.11 | 1.26±0.52 | 0.707 | [4] |
| **EGFR** | 29.43±1.42 | 28.49±1.82 | 0.89±0.26 | 0.740 | [3,5,6] |
| **EIF4E** | 24.99±1.01 | 24.56±1.52 | 1.10±0.10 | 0.511 | [3,7] |
| **GDF5** | **29.00±1.57** | **25.47±1.31** | **0.26±0.08** | **0.005** | **[8]** |
| **IGF1R** | 25.37±1.04 | 24.70±1.37 | 0.93±0.09 | 0.652 | [9] |
| **IRS2** | 25.36±0.88 | 25.07±1.17 | 1.26±0.50 | 0.411 | [10] |
| **KLF4** | 31.23±0.78 | 31.27±1.22 | 1.50±0.31 | 0.233 | [11,12] |
| **MAPKAP1** | **27.95±0.90** | **26.78±1.42** | **0.66±0.05** | **0.009** | **[7]** |
| **MKNK1** | **28.60±0.86** | **27.79±1.46** | **0.85±0.04** | **0.039** | **[7]** |
| **MKNK2** | 25.33±0.90 | 25.31±1.11 | 1.32±0.30 | 0.533 | [7] |
| **MTOR** | 26.43±1.06 | 26.18±1.33 | 1.38±0.43 | 0.465 | [3,13,14] |
| **NR4A3** | 26.74±0.98 | 26.59±0.89 | 1.14±0.46 | 0.862 | [15] |
| **RPS6KB1 (P70S6K)** | 27.67±0.90 | 27.38±1.49 | 1.21±0.04 | 0.098 | [7,13] |
| **PAK1** | 28.31±0.85 | 28.00±1.61 | 1.20±0.10 | 0.182 | [16,17] |
| **PAX6** | 26.97±1.05 | 26.50±1.47 | 1.09±0.15 | 0.627 | [18] |
| **PIK3CD** | 31.95±1.20 | 30.95±1.53 | 0.79±0.16 | 0.312 | [13] |
| **RAF1** | 28.09±0.95 | 27.45±1.41 | 0.91±0.11 | 0.681 | [3,6,19] |
| **PSME3 (REGγ)** | 25.71±1.05 | 25.37±1.55 | 1.18±0.14 | 0.302 | [20] |
| **RELA** | 26.74±0.95 | 26.50±1.51 | 1.27±0.12 | 0.107 | [21,22] |
| **SGK1** | 29.25±0.92 | 28.96±1.39 | 1.16±0.13 | 0.504 | [14] |
| **SMARCD1** | 33.33±0.88 | 32.70±1.30 | 0.94±0.10 | 0.763 | [23] |
| **SNCA** | 29.41±0.68 | 29.06±1.15 | 1.10±0.18 | 0.792 | [24-26] |

**References to supplementary data.**

[1] Oldenburger A, Van Basten B, Kooistra W, et al. (2014) Interaction between Epac1 and Mirna-7 in Airway Smooth Muscle Cells. Naunyn Schmiedebergs Arch Pharmacol 387: 795-797

[2] Saydam O, Senol O, Wurdinger T, et al. (2011) Mirna-7 Attenuation in Schwannoma Tumors Stimulates Growth by Upregulating Three Oncogenic Signaling Pathways. Cancer Res 71: 852-861

[3] Glover AR, Zhao JT, Gill AJ, et al. (2015) Microrna-7 as a Tumor Suppressor and Novel Therapeutic for Adrenocortical Carcinoma. Oncotarget 6: 36675-36688

[4] Hua K, Jin J, Zhang H, et al. (2016) Microrna-7 Inhibits Proliferation, Migration and Invasion of Thyroid Papillary Cancer Cells Via Targeting Cks2. Int J Oncol 49: 1531-1540

[5] Webster RJ, Giles KM, Price KJ, Zhang PM, Mattick JS, Leedman PJ (2009) Regulation of Epidermal Growth Factor Receptor Signaling in Human Cancer Cells by Microrna-7. J Biol Chem 284: 5731-5741

[6] Suto T, Yokobori T, Yajima R, et al. (2015) Microrna-7 Expression in Colorectal Cancer Is Associated with Poor Prognosis and Regulates Cetuximab Sensitivity Via Egfr Regulation. Carcinogenesis 36: 338-345

[7] Wang Y, Liu J, Liu C, Naji A, Stoffers DA (2013) Microrna-7 Regulates the Mtor Pathway and Proliferation in Adult Pancreatic Beta-Cells. Diabetes 62: 887-895

[8] Liu W, Zhang Y, Xia P, et al. (2016) Microrna-7 Regulates Il-1beta-Induced Extracellular Matrix Degeneration by Targeting Gdf5 in Human Nucleus Pulposus Cells. Biomed Pharmacother 83: 1414-1421

[9] Jiang L, Liu X, Chen Z, et al. (2010) Microrna-7 Targets Igf1r (Insulin-Like Growth Factor 1 Receptor) in Tongue Squamous Cell Carcinoma Cells. Biochem J 432: 199-205

[10] Giles KM, Brown RA, Epis MR, Kalinowski FC, Leedman PJ (2013) Mirna-7-5p Inhibits Melanoma Cell Migration and Invasion. Biochem Biophys Res Commun 430: 706-710

[11] Kong B, Wu PC, Chen L, et al. (2016) Microrna-7 Protects against 1-Methyl-4-Phenylpyridinium Iodide-Induced Cell Apoptosis in Sh-Sy5y Cells by Directly Targeting Krupple-Like Factor 4. DNA Cell Biol 35: 217-225

[12] Li YZ, Wen L, Wei X, et al. (2016) Inhibition of Mir-7 Promotes Angiogenesis in Human Umbilical Vein Endothelial Cells by Upregulating Vegf Via Klf4. Oncol Rep 36: 1569-1575

[13] Fang Y, Xue JL, Shen Q, Chen J, Tian L (2012) Microrna-7 Inhibits Tumor Growth and Metastasis by Targeting the Phosphoinositide 3-Kinase/Akt Pathway in Hepatocellular Carcinoma. Hepatology 55: 1852-1862

[14] Qin K, Zhong X, Wang D (2016) Microrna-7-5p Regulates Human Alveolar Epithelial Sodium Channels by Targeting the Mtorc2/Sgk-1 Signaling Pathway. Exp Lung Res 42: 237-244

[15] Stevanato L, Sinden JD (2014) The Effects of Micrornas on Human Neural Stem Cell Differentiation in Two- and Three-Dimensional Cultures. Stem Cell Res Ther 5: 49

[16] Reddy SD, Ohshiro K, Rayala SK, Kumar R (2008) Microrna-7, a Homeobox D10 Target, Inhibits P21-Activated Kinase 1 and Regulates Its Functions. Cancer Res 68: 8195-8200

[17] Yue K, Wang X, Wu Y, Zhou X, He Q, Duan Y (2016) Microrna-7 Regulates Cell Growth, Migration and Invasion Via Direct Targeting of Pak1 in Thyroid Cancer. Mol Med Rep 14: 2127-2134

[18] Needhamsen M, White RB, Giles KM, Dunlop SA, Thomas MG (2014) Regulation of Human Pax6 Expression by Mir-7. Evol Bioinform 10: 107-113

[19] Liu Z, Liu Y, Li L, et al. (2014) Mir-7-5p Is Frequently Downregulated in Glioblastoma Microvasculature and Inhibits Vascular Endothelial Cell Proliferation by Targeting Raf1. Tumour Biol 35: 10177-10184

[20] Shi Y, Luo X, Li P, et al. (2015) Mir-7-5p Suppresses Cell Proliferation and Induces Apoptosis of Breast Cancer Cells Mainly by Targeting Reggamma. Cancer Lett 358: 27-36

[21] Choi DC, Chae YJ, Kabaria S, et al. (2014) Microrna-7 Protects against 1-Methyl-4-Phenylpyridinium-Induced Cell Death by Targeting Rela. J Neurosci 34: 12725-12737

[22] Giles KM, Brown RA, Ganda C, et al. (2016) Microrna-7-5p Inhibits Melanoma Cell Proliferation and Metastasis by Suppressing Rela/Nf-Kappab. Oncotarget 7: 31663-31680

[23] Hong CF, Lin SY, Chou YT, Wu CW (2016) Microrna-7 Compromises P53 Protein-Dependent Apoptosis by Controlling the Expression of the Chromatin Remodeling Factor Smarcd1. J Biol Chem 291: 1877-1889

[24] Doxakis E (2010) Post-Transcriptional Regulation of Alpha-Synuclein Expression by Mir-7 and Mir-153. J Biol Chem 285: 12726-12734

[25] Junn E, Lee KW, Jeong BS, Chan TW, Im JY, Mouradian MM (2009) Repression of Alpha-Synuclein Expression and Toxicity by Microrna-7. Proc Natl Acad Sci U S A 106: 13052-13057

[26] Latreille M, Hausser J, Stutzer I, et al. (2014) Microrna-7a Regulates Pancreatic Beta Cell Function. J Clin Invest 124: 2722-2735
